# Associations between Parenting, Temperament-Related Self-Regulation and the Moral Self in Middle Childhood

**DOI:** 10.3390/children10020302

**Published:** 2023-02-04

**Authors:** Jessica Schütz, Neele Bäker

**Affiliations:** Department of Special Needs Education and Rehabilitation, Carl von Ossietzky University of Oldenburg, Ammerleander Heerstr. 114-118, 26129 Oldenburg, Germany

**Keywords:** moral self, parental warmth, harsh parenting, temperamental self-regulation, special educational needs in emotional–social development, middle childhood

## Abstract

The moral self is increasingly being debated in research, i.e., what causes children to internalise and evaluate the importance of certain moral values. The aim of the present study is to analyse associations between parental warmth and harsh parenting, temperamental self-regulation (inhibitory control and impulsivity), and the moral self in middle childhood. A total of 194 (*n* = 52 children with special educational needs in emotional–social development) six- to eleven-year-old children (*M_age_* = 8.53, *SD_age_* = 1.40) and their primary caregivers (*M_age_* = 40.41, *SD_age_* = 5.94) participated in this cross-sectional questionnaire study. Parental warmth and impulsivity were associated with the moral self. Impulsivity mediated the relationship between harsh parenting as well as parental warmth and the moral self. Results are discussed in terms of their relevance to social information processing theory. The importance of parenting and temperamental self-regulation is discussed as implications that may in turn strengthen children’s moral selves.

## 1. Introduction

Children’s moral self is defined as their individual understanding and integration of moral norms, values, and rules that influence their behaviour and choices in moral situations [1,2,3]. However, the literature highlights that the construct of the moral self is not well understood yet [4,5]. Furthermore, there is a lack of studies analysing the moral self in middle childhood. To better understand the moral self, it seems promising to focus on factors that were already associated with the morality in middle childhood.

For example, some studies suggest that children’s self-regulation is associated with morality [6,7]. Children with higher self-regulatory skills are able to better adapt their behaviour to moral norms and values and have a higher tendency to participate in moral action [8]. A meta-analysis highlighted that in terms of the relationship between self-regulation and morality in childhood, temperament-related aspects of self-regulation, moral behaviour and moral emotions were most frequently analysed [9]. However, a research gap seems to emerge with respect to the moral self [10]. The moral self in middle childhood represents a central developmental phase. The role of authority is increasingly replaced by the child’s own internalised norms. Children in this phase detach themselves from imposed rules and develop their own moral understanding [11]. The underlying mechanisms have not yet been explored [4]. According to Schütz and Koglin [9] temperament-related aspects of self-regulation offer a first empirical approach for linking these constructs. Our study expands this approach to temperament-related aspects of self-regulation and the moral self in middle childhood. In addition to the biologically reactive aspect of temperament-related self-regulation, interactions with emotionally available caregivers also affect children’s moral selves [12]. Studies have highlighted that family structures, parent–child interactions, and parenting styles have been linked to children’s moral selves [13,14,15,16]. Additionally, studies have shown that parenting also affects children’s self-regulatory skills [17,18]. In this study, these constructs will be considered together in an integrative model.

### 1.1. The Moral Self in Middle Childhood

The moral self encompasses the extent to which morality is important to a person’s self-concept [3], for example, how important it is to children to be fair, empathic, or generous [19]. Kochanska [14] defines the moral self as a developing self-concept of children that relates to moral conditions. It is described as an explanatory concept, and also encompasses feelings of personal responsibility [1]. 

Blasi [1,2] emphasises the importance of the moral self in his theory of the self-model. He particularly highlights the importance of the moral self for moral judgement, cognition, and moral action. Other studies also argue that the moral self is essential for moral and prosocial actions. However, the processes responsible for this are not yet clear [3,19,20]. Children evaluate moral conflicts in their everyday lives based on internalised moral values. This evaluation may be influenced by different needs, norms, and orientations. Additionally, the perception of the moral conflict experienced may also vary. In such conflict situations social information processing starts (see SIP model) [21,22]. 

The social information processing (SIP) theory of Crick and Doge [21] and its conceptual development by Lemerise and Arsenio [23] comprise explanatory approaches to the occurrence of aggressive behaviour and conduct disorders. The theory focuses on how children process information in social situations and what happens when there are deficits and biases in the information processing. In the literature such deficits are considered to increase the likelihood of aggressive behaviour [21,23]. Lemerise and Arsenio [23] emphasise the relevance of emotional processes (e.g., emotions and temperament) in the social-cognitive information processing model. Deficits in this area can, for example, contribute to the cognitive process being interrupted due to strong emotional impulses. 

Garrigan et al. [22] and Schipper et al. [24] adapt the SIP model to moral decision making, behaviour, and development. The emotion processes (e.g., emotions and temperament) of Lemerise and Arsenio’s [23] adapted version of the SIP model show associations to the database in which the moral self is to be located [22,24]. The database can be viewed as a repository of resources that are augmented by moral schemas and an understanding of morality. Based on this database, individuals are capable of weighing, deciding, and behaving based on moral principles [24]. Based on a theoretical embedding in social cognitive information processing [21,23] and, considering the extension of emotional component by Lemerise and Arsenio [23], the influence of emotions and temperament on moral development is discussed.

Parenting also plays an important role in the development of children’s moral selves. Children, for example, internalise moral values, rules, norms and what is declared as misbehaviour in their self-concepts through the transmission of parental norms as well as the parent–child relationships [14,15]. 

### 1.2. Parenting 

There is a variety of parenting practices in the literature, which can be categorised into different parenting styles. Research has examined the relationship between parenting styles and child development [17,25,26,27,28]. Baumrind [26], for example, classified four parenting styles: (1) authoritative parenting (high levels of warmth and strictness); (2) authoritarian parenting (restrictive control, punitive parenting practices, low warmth); (3) permissive parenting (low control and high warmth); and (4) neglectful parenting (low control and low warmth [26,27]. 

In recent studies, parental warmth and harsh parenting have often been analysed as opposite poles [17]. Parental warmth includes high-quality or positive parenting and structure, whereas harsh parenting includes rejection but also chaotic and coercive parenting [28]. Studies analysing child development indicate that harsh parenting is not supportive, while parental warmth can have a positive effect on children’s social and emotional development [17,28]. Family structures, parent–child interactions, and parenting styles have also been theoretically and empirically linked to children’s moral selves [13,14,15,16,29]. Parental warmth has already been positively associated with morality in studies with adolescents [30]. Studies emphasises the importance of positive parent–child relationships in the development of children’s moral selves [16,31]. A research gap can be identified with respect to the effects of parental warmth and harsh parenting on children’s moral selves in middle childhood. 

Additionally, several studies have shown that parenting affects children’s self-regulatory skills [17,18]. For example, parental warmth has a positive effect on temperamental self-regulation [32,33] and harsh parenting has a negative effect [33].

### 1.3. Temperamental Self-Regulation

Self-regulatory skills include various abilities to control thoughts, emotions, and behaviour. They develop from early childhood to adulthood. Particularly during kindergarten and preschool age, there is rapid growth in these skills [34,35,36]. Studies use different definitions and operationalisations of self-regulation depending on the research perspective (e.g., temperament research; see Kochanska et al., [37]; cognitive psychology perspectives; see Hinnant et al., [38]). 

Temperament is a biological component and includes individual differences in self-regulation and reactivity [39,40,41,42]. Reactivity is part of the bottom-up regulatory processes and is predominantly automatic. Self-regulation, on the other hand, is goal-directed and conscious, and thus involves top-down processes [35]. It also includes processes of self-soothing and can help modulate reactivity [39,40,41,42]. Studies define and operationalise inhibitory control and impulsivity as temperamental aspects of self-regulation. Inhibitory control encompasses the ability to purposefully suppress dominant stimuli or maladaptive responses [43]. Impulsivity, on the other hand, is less reflective and less intentional. It is a stimulus-driven action that can be adaptive or maladaptive. Both bottom-up processes and top-down processes become active [35]. 

Temperamental processes become relevant for individual differences in personality and may influence the development of different personality traits and social outcomes by interacting with experience [41]. Children’s temperament and their self-concept have already been linked theoretically [40]. However, Kochanska et al. [10] highlighted that further research should analyse associations between inhibitory control and the moral self of children empirically. When considering developmental processes, the question of the interrelationships between temperamental personality traits and self-procured dimensions in middle childhood arises.

Social information processing can be inhibited by deficits in emotion processes like high emotionality, difficult temperament, or deficits in emotion regulation. These deficits can lead to difficulties in accessing information of the database (e.g., moral self, acquired rules, social schemas, or social knowledge; see Arsenio and Lemerise, [44]; Schipper et al., [24]). Furthermore, Hastings et al. [45] discuss that children with agreeable temperament show more concern for others. Lapsley and Hill [46] point out the relevance of further research on the influence of self-regulation on the development of the moral self. 

### 1.4. Current Study 

Parent–child interactions and parenting styles affect children’s development [17,25], their temperamental self-regulation [32,33], and their moral self [13,14,15,16,29]. In addition, temperament-related aspects of self-regulation, moral behaviour and moral emotions were frequently analysed [9]. Unregulated children are more likely to focus on their own needs [45,47]. Nevertheless, there is a research gap for the moral self in middle childhood [4]. The literature has theoretically discussed the fact that children’s temperament affects the development of the moral self-concept [10,41,48]. Additionally, children with difficult temperament or deficits in emotion regulation can be inhibited in social information processing and accessing information of the database (e.g., moral self; Arsenio and Lemerise [44]; Schipper et al. [24]). Nevertheless, empirical results analysing associations between temperamental self-regulation and the moral self in middle childhood are needed.

Moreover, research on parenting, temperamental self-regulation, and the moral self is still needed. 

To better understand the construct of the moral self, this study aims to contribute to the research gap in middle childhood and examines direct and indirect effects between parenting, temperamental self-regulation, and the moral self in an integrative path model. We hypothesise that direct effects of warmth and harsh parenting on the child’s moral self can be mapped. Furthermore, we hypothesise that temperamental self-regulation (inhibitory control and impulsivity) mediates these effects. The terms direct and indirect effect (mediator) were used in the statistical sense, because the study design was cross-sectional.

## 2. Materials and Methods

### 2.1. Participants and Procedure

The present study is a cross-sectional questionnaire study. The data stem from a larger project conducted under the direction of the authors with the goal of examining the social–emotional development of morality in middle childhood. Data were collected between January and May 2022. Only those instruments and data relevant to the current research question are reported. The study was approved by the institutional review board, institutional data protection and information security management, and the relevant regional school authority boards. Randomly selected schools in different regions of Germany were contacted and informed about the study. Firstly, school principals decided whether to participate in the study, and whether data collection would be conducted in school or online. If the school principals agreed to participate, the consent forms were distributed to primary caregivers of students in grades 1 through 4. All participants were informed about the study, the confidentiality of their data and the voluntariness of participation. Only those participants who provided signed consent forms were allowed to participate. A standardised questionnaire was chosen. Primary caregivers report on their parenting style (warmth and harsh parenting) and children’s temperamental self-regulation (impulsivity and inhibitory control). Children report on their moral self. The survey was fully pseudonymised. 

A total of 194 six- to eleven-year-old children (*M_age_* = 8.53, *SD_age_* = 1.40; 58.8% male) and their primary caregivers (*M_age_* = 40.41, *SD_age_* = 5.94) participated in the study. It is an ad hoc sample. A total of 146 children visited regular elementary schools, and 48 children visited a special school. A total of 52 children had special educational needs in emotional–social development (short: SEN). The term special educational needs in emotional–social development is an educational term and comprises conduct disorder, including internalising and externalising behavioural problems [49]. When children in Germany experience developmental or learning impairments in regular school, a procedure for assessing special education needs can be initiated [50]. Exemplary characteristics of children with special education needs in emotional–social development are impairments in perception, behaviour problems [51], lack of empathy and social–emotional skills, higher impulsiveness, and aggressive behaviour [52,53]. As an explanatory approach for the occurrence of aggressive behaviour in this context, the social-cognitive information processing model is often used [21]. Due to the small number of participants that were children with SEN, these results should be interpreted with caution. Accordingly, we added SEN as a control variable in the analysis.

Using G*Power [54], a sensitivity analysis was conducted to determine whether the current study had sufficient power. The post hoc test for the total sample size (*N* = 194) yielded a power of 92.71% when an effect size of *f^2^* = 0.05, α err prob. = 0.05, and seven predictors were included. Despite sufficient statistical power, the results should be interpreted with caution.

### 2.2. Instruments

*Warm and harsh parenting* (EEI; [55]). The parenting style was assessed using the Parenting Style Inventory [55]. Primary caregivers rate their parenting behaviour using a four-point rating scale ranging from (1) strongly disagree to (4) strongly agree. Parental warmth (e.g., “Even in difficult times, I always feel a deep affection for my children”. *a* = 0.89; ten items) and harsh parenting (e.g., “As a matter of principle, one should not discuss rules with children”. *a* = 0.84; ten items) were assessed. The questionnaire has already been psychometrically evaluated and shows adequate validity and reliability [55].

*Inhibitory control and impulsivity* (TMCQ; [56]). Primary caregivers rate children’s temperamental inhibitory control and impulsivity on a five-point Likert scale ranging from (1) Almost always untrue to (5) Almost always true. Inhibitory control includes inhibition skills and is active at the cognitive level (e.g., “Likes to plan carefully before doing something”. *a* = 0.83; eight items). Impulsivity includes non-reflective and stimulus-driven actions where goal-relevant options were also available (e.g., “Cannot help touching things without getting permission”. *a* = 0.89; 13 items; [39,56]. This questionnaire is a valid instrument for measuring temperament [56]. In this study, the focus is on temperament-related self-regulation.

*Moral self* [57]. To assess the moral self of the children, we used the version adapted for children of the questionnaire developed by Koglin [56] to measure moral identity in adolescence. The questionnaire has shown adequate reliability in prior studies with adolescents [24]. The questionnaire was designed with respect to the Self-Importance of Moral Identity Measure [19] and the adapted version of the Good Self-Assessment [58]. The moral self scale included 18 items. The children rated the personal relevance of nine moral characteristics (e.g., being compassionate or selfless) and nine non-moral characteristics (distractors e.g., being funny or smart) on a four-point rating scale ranging from (1) not at all important to me to (4) very important to me (*a* = 0.71; nine items).

### 2.3. Data Analytic Procedures

SPSS 27 and AMOS 27 were used for data analysis. First, the data were analysed descriptively. Then, the postulated path model was analysed. The path model included parental warmth and harsh parenting as independent variables, inhibitory control and impulsivity as mediator variables, and children’s moral selves as the dependent variable. Gender, age, and special educational needs in emotional–social development (dummy coded: gender was coded with girls = 1 and boys = 2; SEN was coded with 1 = without SEN and 2 = with SEN) were controlled as additional independent variables. The terms dependent variable, independent variable, mediator, and direct or indirect effect were used in the statistical sense, because the study design was cross-sectional. In addition to direct effects between variables, indirect mediation effects were also estimated using a bootstrap method with confidence estimates (95% level confidence interval; bootstrap of 1000 samples; [59]). Missing data were tested for the Missing Completely at Random condition (*MCAR*; *χ^2^* = 51.788, *df* = 44, *p* = 0.196; [60]) and subsequently estimated using Full Information Maximum Likelihood estimation of the regression coefficient. To evaluate the model fit of the postulated path model, the following cut-off criteria were used for interpretation: *χ^2^* not significant, *χ^2^/df* < 5, *CFI*, *NFI*, and *TLI* close to 1 (>0.90), and *RMSEA* close to 0 (<0.08) [61,62].

## 3. Results

Descriptive statistics of each variable, as well as the intercorrelations between them, are presented in Table 1. Mean values of the variables indicated that level of parental warmth, inhibitory control and the moral self were high and harsh parenting and impulsivity were rated as being average. Cronbach’s alpha and McDonald’s omega indicated sufficient reliability for the variables [63,64]. Parental warmth correlated positively with inhibitory control and the moral self, and negatively with impulsivity and SEN. Harsh parenting correlated positively with gender and negatively with SEN. Inhibitory control correlated negatively with impulsivity, the moral self, gender, and SEN. Impulsivity was only correlated negatively with the moral self and positively with gender and SEN. 

**Figure 1:**
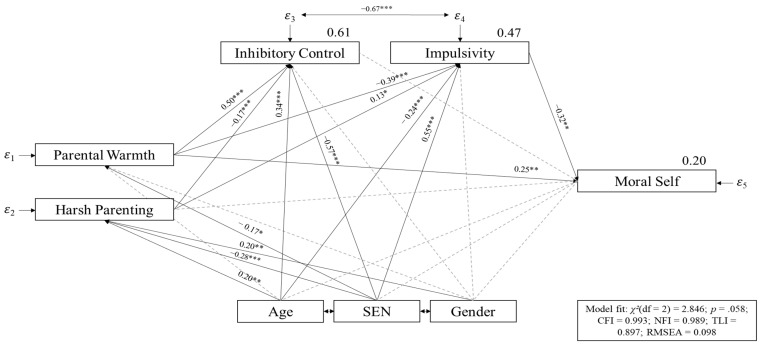
Path model. Note. * *p* < 0.05; ** *p* < 0.01; *** *p* < 0.001; values above the variable names indicate explained variance; non-significant paths were marked with grey dashed lines, SEN = special educational needs in emotional–social development, gender was coded with girls = 1 and boys = 2; SEN was coded with 1 = without SEN and 2 = with SEN.

Figure 1 shows the tested path model with significant direct effects. The model revealed an adequate model fit (*χ^2^*(*df* = 2) = 2.85; *p* = 0.06; *CFI* = 0.99; *NFI* = 0.99; *TLI* = 0.90; *RMSEA* = 0.10). Twenty percent of the variance of the moral self, 61% of the variance of inhibitory control, and 47% of the variance of impulsivity was explained. Regarding the direct effects, only parental warmth was positively related, and impulsivity was negatively related, to the moral self (warmth *β* = 0.25, *p* = 0.003; impulsivity *β* = −0.32, *p* = 0.007). Parental warmth was positively related to inhibitory control (*β* = 0.50, *p* < 0.001) and negatively to impulsivity (*β* = −0.39, *p* < 0.001). Harsh parenting was negatively related to inhibitory control (*β* = −0.17, *p* < 0.001), and positively to impulsivity (*β* = 0.13, *p* = 0.020). 

Age was associated positively with harsh parenting (*β* = 0.20, *p* = 0.007), inhibitory control (*β* = 0.34, *p* < 0.001) and negatively with impulsivity (*β* = −0.24, *p* < 0.001). Gender was only associated positively with harsh parenting (*β* = 0.20, *p* = 0.005). Further associations with special educational needs in emotional–social development and warmth (*β* = −0.17, *p* = 0.031), harsh parenting (*β* = −0.28, *p* < 0.001), inhibitory control (*β* = −0.57, *p* < 0.001) and impulsivity (*β* = 0.55, *p* < 0.001) were identified.

There were also indirect effects between warmth (*β* = 0.13, *p* = 0.007) and harsh parenting (*β* = −0.04, *p* = 0.036) and the moral self, mediated by impulsivity. 

## 4. Discussion

The aim of the present study was to analyse the associations between parenting, temperamental self-regulation, and the moral self in an integrative path model to better understand the moral self in middle childhood. Therefore, direct and indirect effects between parenting, temperamental self-regulation, and the moral self were examined. 

The results suggest that impulsivity was negatively related to the moral self. The importance of temperament-related self-regulation has already been discussed in the literature for moral emotions and behaviour [9]. The current study addresses this issue by emphasising the importance of temperament-related self-regulation for the moral self. In the literature, some indications that children’s temperament and self-regulatory skills are important for their moral selves can be found. Lemerise and Arsenio [23], for example, highlight that emotion processes (e.g., regulation and temperament) in social information processing [21] show direct effects on the database in which the moral self is located [22,24,44]. The results of the current study support this theory. Children who are impulsive may have difficulty internalising or retrieving moral schemata and values in social contexts [22]. Children who tend to be impulsive may have a weak ability to delay satisfaction [46]. It could be assumed that these children consequently have difficulties focusing on the concern of others. They do not internalise or focus on moral values like empathy, justice, and fairness to others because these values could conflict with their own needs in moral situations. 

Furthermore, Kochanska et al. [10] discuss that moral behaviour in prior situations could mediate the relationship between the child’s temperamental self-regulation and their moral self. Children who act impulsively tend to focus on their own need satisfaction rather than focusing on the needs of others [45,47]. This is also transferable to moral conflicts. In moral conflicts, the own needs compete with the needs of others. It is conceivable that children who act impulsively in moral conflicts may experience a short-term reward (e.g., need satisfaction or escape from the stressful situation) by focusing on their own needs rather than the needs of others. This can lead to immoral behaviour and, at the same time, a short-term reward. This short-term reward could ensure that the internalisation of moral values is inhibited. From moral or immoral behaviour, children in turn can learn what is important to them in moral conflicts. Further studies should analyse the internalisation of moral values into the moral self in early childhood in relation to temperamental self-regulation and moral behaviour. 

Parental warmth was positively associated with the moral self. This result is consistent with studies associating positive parenting with children’s moral development [13,14,15,16,29,30,31]. If children experience warmth and affection from their caregivers, they internalise values of kindness and love into their own self-concept, and therefore in their moral self. Sengsavang and Krettenauer [16] also discuss that positive parenting, such as parental support, may be reflected in children’s moral behaviour. If children have experienced support, they have also internalised moral values such as support into their moral selves, so that they tend to act supportively in moral situations. 

In the correlation, parental warmth and the two temperament-related aspects of self-regulation (inhibitory control and impulsivity) correlate with the children’s moral selves. In the path model, only parental warmth and impulsivity were associated with the moral self. This could indicate a redundancy effect [65,66]. It can be assumed that impulsivity and inhibitory control explain the same variance in the moral self. Moreover, the two variables are strongly correlated, and impulsivity prevailed due to the stronger direct effect in the path model. Nevertheless, results support the importance of temperament-related self-regulatory for children’s moral selves.

Additionally, a suppression effect can be assumed. Harsh parenting did not correlate with inhibitory control and impulsivity, but in the path model, harsh parenting was significantly related to inhibitory control and impulsivity [67]. In the correlations, parental warmth had a significant effect on inhibitory control and impulsivity. In this sample, harsh parenting was associated with more impulsivity and less inhibitory control, which corresponds to low self-regulation. Parental warmth was associated with impulsivity and more inhibitory control, which corresponds to a high degree of self-regulation. These results are consistent with findings that showed that parenting is associated with children’s self-regulatory skills [17,18]. Parental warmth is positively [32,33] and harsh parenting is negatively related to temperamental self-regulation [33]. Eisenberg et al. [32] argue that parental warmth has been associated with secure attachment [68], which in turn fosters regulated behaviour in children [69].

Furthermore, impulsivity emerged as a mediator of the relationship between harsh as well as warm parenting and the moral self. The mediating role of impulsivity emphasises the promotion of self-regulatory skills and impulse control for the development of the moral self in middle childhood. A practical implication that further arises is to foster positive parenting characterised by warmth, acceptance, and structure since this in turn supports both children’s impulse control (lower impulsivity) and their moral selves. 

Age, gender, and SEN were also controlled in the path model. With increasing age, harsh parenting and inhibitory control also increasing, while impulsivity decreased. Gender was only associated with harsh parenting at the expense of the boys. SEN were associated with harsh and warm parenting, inhibitory control and impulsivity. Children with SEN experienced more harsh parenting and showed higher levels of impulsivity and lower levels of inhibitory control. These findings are consistent with literature reporting that children with special educational needs in emotional–social development are more likely to show difficulties in self-regulation (behavioural, attentional, cognitional, and emotional regulation; [70,71,72]. Harsh parenting and behaviour problems are also increasingly being associated in the literature [73]. 

### Limitations and Further Research 

Although a strength of the current study is that new directions of the moral self are discussed and the results address the research gap on the moral self in middle childhood [4], some limitations should also be noted. Methodological limitations include the ad hoc sample and that the questionnaire assessing children’s moral selves [57] having not yet been psychometrically evaluated. However, the reliability coefficients in the current study were adequate. Additionally, the model fit index RMSEA was quite high. This could be due to the complexity of the model. However, overall, the model shows an adequate fit. Another methodological limitation is the cross-sectional design. To examine the reciprocal effects and the influence of parenting and temperamental self-regulation in the development of children’s moral selves, longitudinal designs are needed. 

The number of children with special educational needs in emotional–social development in the current study was very small (*n* = 52). However, the results show promising approaches for further research. The question arises as to which initial situations children with special educational needs in emotional–social development have that inhibit them in their moral development. It is possible that children with SEN internalise similar moral values into their self-concept, but show deficits in relevant antecedent skills. These inhibit them in social information processing in moral contexts and ensure that they have difficulty retrieving their moral values in such situations. Further research could analyse characteristics associated with the special educational needs in emotional–social development in relation to moral development. Studies also argue that the moral self [3,20], parenting [74], and children’s self-regulatory skills [75] are essential for moral and prosocial actions. In addition to the analysed constructs in predicting moral self, all three constructs are shown to be predictors of moral and prosocial behaviour in independent studies. In view of this, a joint consideration of all constructs would be of scientific interest.

## 5. Conclusions

This study highlights the importance of impulsivity and harsh as well as warm parenting for the moral self in middle childhood, and therefore addresses the research gap highlighted by Kingsford et al. [4] and Schütz and Koglin [9]. Furthermore, the results support the theory that emotional processes (e.g., regulation and temperament) in social information processing [21] show associations to the database in which the moral self is located [22,23,24]. Preventions and interventions could focus on parental warmth and foster temperamental self-regulation that may in turn strengthen children’s moral selves. Additionally, further research should analyse the internalisation of moral values into the moral self in early childhood in relation to temperamental self-regulation and moral behaviour.

## Figures and Tables

**Table 1:** Intercorrelations, means, standard deviations and reliability.

	1	2	3	4	5
1 Parental warmth	1				
2 Harsh parenting	0.14	1			
3 Inhibitory control	0.55 ***	0.02	1		
4 Impulsivity	−0.45 ***	−0.04	−0.83 ***	1	
5 Moral self	0.35 ***	−0.00	0.35 ***	−0.38 ***	1
6 Age	−0.04	0.12	0.07	0.01	0.07
7 Gender	0.01	0.16 *	−0.18*	00.16 *	−0.03
8 SEN	−0.15 *	−0.16 *	−0.51 ***	0.52 ***	−0.14
*M*	36.79	26.14	27.33	36.59	31.64
*SD*	3.28	4.33	6.21	8.98	3.01
*α*	0.89	0.84	0.83	0.89	0.71
Ω	0.90	0.84	0.82	0.89	0.70

Note. * *p* < 0.05; *** *p* < 0.001; *M* = mean; *SD* = standard deviation, α = Cronbach’s alpha, Ω = McDonald’s Omega; SEN = special educational needs in emotional–social development; gender was coded with girls = 1 and boys = 2; SEN was coded with 1 = without SEN and 2 = with SEN.

## Data Availability

The data that support the findings of this study are available on request from the corresponding author. The data are not publicly available due to privacy or ethical restrictions.
